# the Safety of Pulmonary Ultrasonography in the Neonatal Intensive Care Unit

**DOI:** 10.34763/devperiodmed.20182201.7580

**Published:** 2018-04-12

**Authors:** Mateusz Jagła, Olga Krzeczek, Aleksandra Buczyńska, Zuzanna Zakrzewska, Przemko Kwinta

**Affiliations:** 1 Chair of Pediatrics, Jagiellonian University, Collegium Medicum, Cracow, Poland; 2 Student Research Group, Chair of Pediatrics, Jagiellonian University, Collegium Medicum, Cracow, Poland

**Keywords:** pulmonary capillary hemorrhage, lung ultrasound, very low birth weight infant, krwotok płucny, ultrasonografia płuc, bardzo niska masie urodzeniowej

## Abstract

**Introduction:**

Due to specific anatomy of children are more vulnerable to the carcinogenic effects of ionizing radiation from chest X-rays. Lung ultrasound (LUS) is a validated procedure which can easily be used in diagnosing pathologies of the neonatal lung. However, experimental studies have shown that low frequency ultrasound may induce pulmonary capillary hemorrhage (PCH).

**Aim of the study:**

To evaluate the potential relationship between lung ultrasound and pulmonary hemorrhage in very low birth weight infants.

**Patients and methods:**

We analysed the medical records of very low birth weight infants admitted to our neonatal tertiary centre between 2008 and 2011 (group 1), when CXR was the main procedure used to evaluate the respiratory system, and between 2013 and 2016 (group 2), when LUS became a routine procedure, replacing the chest X-ray.

**Results:**

297 infants were enrolled in the first group and 286 in the second group, respectively. There was no difference in the frequency of pulmonary hemorrhages between the two groups (p=1). In the first group there was only one episode of PCH and in the second group no PCH was seen. Statistically significant differences were seen in a number of patients with pulmonary hemorrhage risk factors: surfactant administration (p<0.001), mechanical ventilation (p=0.0003), and hemodynamically significant patent ductus arteriosus (p=0.025).

**Conclusions:**

Routine lung ultrasound appears to be safe in very low birth weight infants; there were no episodes of pulmonary hemorrhage.

## Streszczenie

**Wprowadzenie:**

Swoista budowa anatomiczna ustroju dziecięcego może zwiększać podatność na działanie rakotwórcze promieniowania jonizującego wykorzystywanego do wykonywania zdjęć przeglądowych klatki piersiowej (CXR). Alternatywną metodą diagnostyczną stosowaną u noworodków jest ultrasonografia płuc (LUS). Wyniki badań eksperymentalnych wykazały jednak, że ultradźwięki o niskiej częstotliwości mogą powodować krwotoki kapilarne płuc (PCH).

**Cel badania:**

Ocena potencjalnej zależności pomiędzy ultrasonografią płuc a krwotokiem płucnym u noworodków z bardzo małą masą urodzeniową.

**Pacjenci i metody:**

Przeprowadzono analizę dokumentacji medycznej noworodków o bardzo małej masie urodzeniowej, przyjętych w latach 2008-2011 (grupa 1), gdy zdjęcie przeglądowe klatki piersiowej było zasadniczą procedurą stosowaną do oceny układu oddechowego, oraz w latach 2013-2016 (grupa 2), kiedy wiodącą metodą była ultrasonografia płuc.

**Wyniki:**

W badaniu nie stwierdzono różnicy w częstości krwotoków płucnych pomiędzy grupami (p=1). W pierwszej grupie był tylko jeden epizod krwotoku płucnego, w drugiej grupie nie obserwowano żadnego przypadku. Stwierdzono istotne statystycznie różnice w częstości czynników ryzyka PCH: leczenie surfaktantem (p<0,001), wentylacji mechanicznej (p=0,0003) i obecności istotnego hemodynamicznie przewodu tętniczego (p=0,025).

**Wnioski:**

Ultrasonografia płuc jest bezpieczną metodą diagnostyczną, w trakcie jej rutynowego stosowania nie stwierdzono zwiększenia częstości krwotoków płucnych.

## Introduction

Lung diseases are some of the most common and serious complications of prematurity [1]. Chest X-rays (CXRs) have remained the most common radiological procedure used in the evaluation of the respiratory system in the neonatal population [2, 3]. Neonates’ increased sensitivity associated with their anatomical features and long life expectancy results in greater vulnerability to the carcinogenic effects of ionizing radiation from chest X-rays [4, 5, 6, 7, 8]. It is also worth emphasizing that chest X-rays are specific, but lack sensitivity. Lung ultrasound (LUS) is another validated procedure which can easily be used in diagnosing pathologies of the neonatal lung. DA Lichtenstein, the forerunner of this useful method, explains its positive qualities, such as bedside diagnosis, avoidance of irradiation, and cost-effectiveness [9]. The use of the lung ultrasound in neonatal intensive care units is increasing because of its high sensitivity and specificity in comparison to chest X-rays in many clinical settings [9, 10]. Nevertheless, it has not been routinely used for diagnosing and controlling neonatal pulmonary diseases [11]. However, as the role of this procedure has begun to grow and take the place of traditional X-rays, clinicians started to ask questions about its safety.

Experimental studies have proven that the range between 1.5-12.0 MHz of ultrasound frequency may induce pulmonary capillary hemorrhage (PCH) in mammals [11, 12, 13, 14, 15]. Particular histologically verified studies were performed among rats. PCH is defined pathologically as the presence of erythrocytes in the pulmonary alveoli, septa, or both. Clinically, it is depicted by bloodstained frothy secretions aspirated from the trachea [16]. Severity of illness, intrauterine growth restriction, patent ductus arteriosus, coagulopathy, and the need for assisted ventilation are risk factors which predispose to PCH. Therefore, these conditions should be taken into consideration while investigating the impact of LUS on inducing PCH.

Our study was conducted at the neonatal intensive care unit, where since 2013 lung ultrasonography has become the leading diagnostic tool in pulmonary disorders in neonates. The increased use of this method, including in neonatal ambulances, has been primarily due to its safety, non-invasiveness, and easy accessibility.

The aim of this study was to find the potential relationship between lung ultrasound and pulmonary hemorrhage in order to evaluate the safety of this method in very low birth weight infants.

## Patients and methods

### Patients

We conducted a retrospective medical records analysis of infants admitted to the Neonatal Intensive Care Unit in the Department of Pediatrics, Jagiellonian University Collegium Medicum, a third level neonatal centre in Cracow, Poland. The Unit is a referral neonatal center located in the southeastern region of Poland. All neonates are transported from other hospitals, because there is no maternity department. Infants were enrolled into the study if their birth weight was <1500g. We divided them into two groups based on their date of admission. The first group includes patients admitted between 2008 and 2011, when chest X-ray was the main procedure used to evaluate the respiratory system. The second group consists of patients admitted between 2013 and 2016, when lung ultrasound became a routine procedure, replacing the chest X-ray. The indications to perform CXR or LUS examination were for example: respiratory failure, suspicion of respiratory distress syndrome, air leak syndromes, congenital pneumonia, lung and diaphragm malformations, and confirming the position of a central venous catheter. In 2012 the transitional time was omitted. Our aim was to compare these two groups and find the potential relationship between the amount of X-ray/USG tests performed and the frequency of PCH.

## Methods

For CXR, the Polymobil Plus (Siemens Healthcare, Germany) analog mobile X-ray machine was used. Lung ultrasound was done on the Philips HD 11 or the Philips Envisor machine equipped with a broadband probe with a frequency of 5-12 MHz (Philips, US).

### Statistical analysis

Fisher’s exact test and Mann-Whitney U tests were used to compare baseline and outcome variables, as appropriate. The results are presented as numbers (percentages) or medians (interquartile range), unless otherwise indicated. Analysis was done using the MedCalc Statistical Software version 16.8.4 (MedCalc Software bvba, Ostend, Belgium; https://www.medcalc.org; 2016). Probability values below 0.05 were considered statistically significant.

## Results

297 infants were enrolled in the first group (2008-2011) and 286 infants were enrolled in the second group (2013-2016). Demographic and clinical characteristics of the patients are included in Table I. Statistically significant differences were observed in mean birth weight (p=0,0027), median gestational age (p=0,0003), median of Apgar scores at five minutes (p=0,0027), number of intraventricular hemorrhages grade III or IV (p<0,001), and the number of deaths before discharge (p=0,021).

**Table I:** Demographic and clinical characteristics of the patients. Values were expressed as numbers (percentages: %) or medians (interquartile range, IQR). Differences between the groups were compared using either the Mann-Whitney U test (1) or Fisher’s exact test (2). Tabela I. Demograficzna i kliniczna charakterystyka pacjentów. Wartości wyrażono jako liczby (procenty, %) lub mediany (przedział kwartylowy, IQR). Różnice pomiędzy grupami badano testem U Manna-Whitney’a (1) lub dokładnym testem Fishera (2).

Variables *Zmienne*	Group 2008-2011 n=297 *Grupa 2008-2011 n=297*	Group 2013-2016 n=286 *Grupa 2013-2016 n=286*	p value *wartość p*
Birth weight, g, median (IQR) *Masa* *ciała, g*, *mediana* ** *(IQR)*	1000 (737.5-1300)	1115 (850-1300)	0.0027^1^
Male gender, n (%) *Płeć męska*, *n* *(%)*	144 (48.3%)	154 (53.8%)	0.21^2^
GA, wk median (IQR) *Wiek* *ciążowy*, *mediana* *(IQR)*	28 (26-30)	29 (26-31)	0.0003^1^
SA ** 5, median (IQR) *Skala Apgar w* *5 min, mediana (IQR)*	6 (5-7)	7 (5-8)	0.0027^1^
IVH III/IV, n (%) *Krwawienie* *dokomorowe III/IV, n (%)*	77 (25.9%)	27 (9.4%)	<0.001^2^
BPD, n (%) *Dysplazja* *oskrzelowo-płucna, n (%)*	68 (22.9%)	54 (18.9%)	0.263^2^
LOH, median (IQR), *Czas* *hospitalizacji*, *mediana (IQR)*	55 (31-89)	47 (32-72)	0.063^1^
Death before discharge, n (%) *Zgon przed* *wypisem*, *n (%)*	27 (9.1%)	12 (4.2%)	0.021^2^

GA – gestational age, *wiek ciążowy*; SA – Apgar score, *skala Apgar*; IVH – intraventricular hemorrhage, *krwawienie dokomorowe*; BPD – bronchopulmonary *dysplasia, dysplazja oskrzelowo-płucna*; LOH – length of hospitalization, *czas hospitalizacji* ; IQR − interquartile range, *przedział kwartylowy*.

**Fig. 1:**
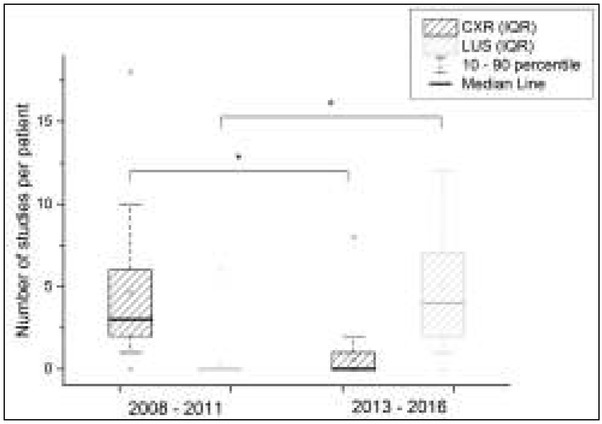
Number of CXR and LUS studies per patients in group 1 (2008-2011) and group 2 (2013-2016) Differences between the groups were compared using the Mann-Whitney U test. Ryc. 1. Liczba zdjęć radiologicznych klatki piersiowej i przezklatkowego badania ultrasonograficznego klatki piersiowej na jednego pacjenta w grupie 1 (2008-2011) i 2 (2013-2016). Różnice pomiędzy grupami badano testem U Manna-Whitney’a.

The analysis of numbers in CXR and LUS studies (Figure 1) revealed a statistically significant difference between groups (p<0.001). Moreover, it showed that over 70% of the patients from the second group (years 2013-2016) did not have any X-rays taken during hospitalization.

There was no difference in the frequency of pulmonary hemorrhages between the two groups. In the first group (2008-2011) there was only one episode of PCH and in the second group (2013-2016) no PCH was seen. Statistically significant differences were seen in a number of patients with pulmonary hemorrhage risk factors (Table II). These risk factors were those who had surfactant administered (p<0.001), those in whom mechanical ventilation was used (p=0.0003), and those who had a hemodynamically significant patent ductus arteriosus (p=0.025).

**Table II:** Analysis of risk factor for pulmonary hemorrhage. Differences between the groups were compared using Fisher’s exact test. Tabela II. Analiza czynników ryzyka wystąpienia krwotoku płucnego. Różnice pomiędzy grupami badano dokładnym testem Fishera.

	Group 2008-2011 n=297 *Grupa 2008-2011 n=297*	Group 2013-2016 n=286 *Grupa 2013-2016 n=286*	p value *wartość p*
Pulmonary hemorrhage, n (%) *Krwotok z płuc, n (%)*	1 (0.3%)	0 (0.0%)	1.0
RDS, n (%) *Zespół zaburzeń oddychania, n (%)*	238 (80.1%)	220 (76.9%)	0.365
Surfactant, n (%) *Surfaktankt, n (%)*	109 (36.7%)	147 (51.4%)	<0.001
Mechanical ventilation, n (%) *Wentylacja mechaniczna, n (%)*	232 (78.1%)	185 (64.7%)	0.0003
FFP transfusion, n (%) *Przetoczenie osocza świeżo mrożonego, n (%)*	76 (25.6%)	68 (23.8%)	0.632
Thrombocytopenia, n (%) *Małopłytkowość, n (%)*	13 (4.4%)	11 (3.8%)	0.836
HsPDA, n (%) *Przetrwały przewód tętniczy, istotny hemodynamicznie, n (*%)	123 (41.4%)	92 (32.2%)	0.025

RDS – respiratory distress syndrome, *zespół zaburzeń oddychania*; FFP – fresh frozen plasma, *osocze świeżo mrożone*; HsPDA − hemodynamically significant patent ductus arteriosus, *przetrwały przewód tętniczy istotny hemodynamicznie*.

## Discussion

Through the development of new neonatal intensive care unit diagnostic techniques, the survival of premature infants was observed to increase. These techniques have put chronic lung disease as the primary cause of mortality, instead of prematurity [17, 18, 19]. Both chronic lung disease of infancy and acute newborn respiratory disorders result from preterm neonates’ lung immaturity. Both prenatal and early postnatal factors may substantially impact the development of consecutive stages of the airways, alveolarization, and forming pulmonary vessels [18]. Susceptibility to respiratory distress syndrome, pulmonary hemorrhage, transient tachypnea of the newborn, pneumonia, and pulmonary hypertension are strongly correlated with abnormalities in the early growth and development of the human lung [21]. One of the most important long-term complications of prematurity is bronchopulmonary dysplasia (BPD), which may result in chronic obstructive pulmonary disease in the latter years of life [21, 22, 23]. The chest X-ray (CXR) has still remained the standard and most frequently performed radiological diagnostic procedure used in the diagnosis of such lung disorders. Unfortunately, this procedure requires the use of radiation, which has the potential to harm infants’ cells. Studies based on cytokinesis-block micronucleus cytome assay with the application of dosimetry systems and cytogenetic status in the child population show that even low dose-diagnostic X-ray exposure may induce damaging effects on infants’ somatic DNA [4]. The main biological effect of X-rays results from the ionization of water molecules, thus forming hydroxyl radicals that may damage DNA [24]. During pregnancy, exposure to radiation should be minimized and benefits should be weighed against possible risks. The radiation sensitivity of a developing fetus depends on the gestational age at the time of exposure [25]. Prenatal death, intrauterine growth retardation, mental retardation, organ malformation, and childhood cancers are possible effects of ionizing radiation depending on the dose and time of exposure during pregnancy [24, 25]. At earlier gestational ages, the fetus has a higher sensitivity to radiation [17]. Preterm infants have more remaining years of life during which a radiation-induced cancer might develop and are 10 to 15 times more radiosensitive than adults [4, 17, 24]. The radiosensitivity of infants was further supported by the results of Turan et. al, whose study focused on exposure to scattered radiation from radiographic examinations occurring in the same room [26]. The impossibility of bedside X-ray assessment during the procedure is another disadvantage.

The goal of the pediatrician is to assess their patients’ lungs without the risk of procedural side effects. Lung ultrasonography is the best alternative to common chest X-rays, due to its ease of learning, accessibility, high sensitivity and specificity. The overall short length of preforming and interpreting LUS allows a bedside evaluation of the patient without exposure to ionizing radiation and frequent repeatability without risk [27, 28]. The anatomical features of infant lungs, such as their thinner thoracic walls and smaller width of the thorax and lung volumes improve the quality of lung surface visualization compared to the adult [29]. Owing to Lovrenski’s prospective study in which respiratory distress syndrome was diagnosed with LUS, there was better reliability in monitoring the clinical changes, as well as more accuracy in the detection and localization of the pathologies in lungs using LUS in comparison to CXRs [17]. The simplicity of the procedure is shown by Badetti et al., in which inexperienced physicians were given 30 minutes of training and were then able to identify pulmonary pathologies in 10 examinations using LUS [30, 31].

Although there are reports pointing out the effectiveness of LUS, there are some experimental studies that question its safety. Douglas L. Miller proved that the range of 1.5-12.0 MHz ultrasound frequencies may induce pulmonary capillary hemorrhage in mammals [32]. The physical mechanisms of forming PCH and dosimetry have not yet been clearly defined. Heating and cavitation are the main phenomena responsible for the biological effects of ultrasound. Tests for heating have contradicted such explanation of ultrasound-induced PCH [33, 34]. Also tests for cavitation did not support the hypothesis of its involvement in PCH [35, 36]. It has been proved that the magnitude of lesions decrease with increasing acoustic impedance difference between the intercostal tissue and the lung [14]. Additionally, the magnitude of PCH decreases with increasing frequency [13]. Alveolar hemorrhage also depends on the volume of lung inflation. The intensity of the lesions is inversely related to the volume of inspired air [14]. In lungs inflated over the tidal volume PCH was not generated, however, the opposite was seen in less inflated lungs. Unlike studies performed on animals, Meltzer et. al preformed studies based on intraoperative transoesophageal echocardiography in humans, with the lowest frequency being 3.5 MHz and the mean age of patients being 61. The results of the Meltzer et. al study did not support the hypothesis of ultrasound as a cause of PCH and confirmed the statement that human lungs are not as sensitive as other mammals’ [37].

In our Neonatal Intensive Care Unit, in which LUS is currently the method of choice to evaluate the respiratory system in the neonatal population, practitioners had an opportunity to verify the usefulness of LUS in their everyday practice. The safety of LUS is supported by observing our unit in which no episodes of pulmonary hemorrhage were seen. Even though all risk factors were present in this patient population, it did not increase the rate of pulmonary hemorrhage.

Lung ultrasound appears to be an improved diagnostic tool for infants’ pulmonary disorders. However, we should not overlook that it has its limitations as with every electronic device. Some pathologies, such as interstitial edema, cannot be detected by LUS [17]. It is important to keep in consideration the length of the examination due to its impact on the behavior of infants. Patient tolerance varies and long procedures may result in fear and unnecessary stress. Patience and precision is vital while preforming the procedure.
